# Baseline predictions of PACC progression trajectories in preclinical AD improve the precision and power of treatment effect assessments

**DOI:** 10.1002/alz70859_099560

**Published:** 2025-12-25

**Authors:** Viswanath Devanarayan, Yuanqing Ye, Michael C. Donohue, Reisa A. Sperling, Keith A. Johnson, Rema Raman, Paul S. Aisen, Lynn D Kramer, Michael C. Irizarry, Shobha Dhadda

**Affiliations:** ^1^ Eisai Inc., Nutley, NJ USA; ^2^ University of Illinois Chicago, Chicago, IL USA; ^3^ Alzheimer's Therapeutic Research Institute, University of Southern California, San Diego, CA, USA, San Diego, CA USA; ^4^ Center for Alzheimer Research and Treatment, Department of Neurology, Brigham and Women’s Hospital, Boston, MA USA; ^5^ Departments of Neurology and Radiology, Massachusetts General Hospital, Harvard Medical School, Boston, MA USA; ^6^ Alzheimer's Therapeutic Research Institute, University of Southern California, San Diego, CA USA; ^7^ Alzheimer's Therapeutic Research Institute, Keck School of Medicine, University of Southern California, San Diego, CA USA

## Abstract

**Background:**

Variability in cognitive decline in preclinical Alzheimer’s disease (AD) presents a significant challenge in evaluating treatment effects in clinical trials. This study developed models to forecast cognitive progression and assessed their potential to improve the precision of treatment effect estimates in future trials.

**Method:**

Data were from the Phase III Anti‐Amyloid Treatment trial of Solanezumab in amyloid‐positive asymptomatic preclinical AD. Due to no significant cognitive decline differences between groups, the Solanezumab arm (n=549) was used for training, and the placebo arm (n=559) for validation. Cognitive decline was assessed as 24‐week changes in Preclinical Alzheimer's Cognitive Composite (PACC) score over 216 weeks.

A predictive model for PACC decline was developed using demographics, APOE ε4 status, and baseline clinical assessments (PACC composite score, its components, and CDR‐SB), employing Stochastic Gradient Boosting. The added value of amyloid PET Centiloid (CL), plasma pTau217 (electrochemiluminescence assay), MRI morphometrics, and tau PET measures (375‐participant substudy) were evaluated. Model performance was optimized via cross‐validation and validated in the placebo group. The impact of baseline PACC decline predictions as an Alzheimer’s Prognostic Covariate (APC) on treatment effect assessments was evaluated in trial simulations.

**Result:**

A model with demographics, CL, and clinical assessments explained 16% of PACC decline at week 216 (R²=0.16), improving over CL or clinical assessments alone (p<0.05). Adding MRI increased R² to 0.23 (p<0.05); replacing CL with plasma pTau217 or tau PET raised it to 0.25 and 0.42, respectively. Without tau PET in the model, key baseline predictors were pTau217, CL, PACC, MMSE, inferior temporal area, and entorhinal volume. When tau PET was included, it became the strongest predictor, with fusiform, inferior temporal, and supramarginal regions being the most predictive.

Simulations showed baseline prediction of PACC decline as APC can reduce treatment effect variance by 20.3%, increase power from 80% to 88%, or reduce sample size by 21.5%. Incorporating tau PET further reduces variance by 37%, increases power to 94%, and decreases sample size by 35%.

**Conclusion:**

Baseline tau PET was the strongest predictor of PACC decline. Using baseline‐predicted PACC decline as APC can enhance treatment effect estimates and trial efficiency in preclinical AD.

